# On the Post-Partem Use of Ergot in Obstetrical Practice

**Published:** 1877-03

**Authors:** Wm. Abram Love

**Affiliations:** Atlanta; Professor of Physiology in the Atlanta Medical College, etc.


ATLANTA

MEDICAL AND SURGICAL JOURNAL

VOL. XIV.] MARCH—1877. [No. 12

### Original Communications.

### ON THE POST-PARTEM USE OF ERGOT IN OBSTETRICAL PRACTICE.

BY WM. ABRAM LOVE, M.D.,
Professor of Physiology in the Atlanta Medical College, etc.

The operation of ergot upon the pregnant uterus is to produce a constant, unremitting contraction of its muscular walls, resulting in an unyielding, continuous rigidity, rather than the alternate contractions and relaxations incident to normal labor. Such physiological action of ergot has long since assigned it a place among our therapeutic agents, beyond which it has not passed, in the hands of most practitioners. Regarded by many as an agent to be used solely in aiding or hastening the process of parturition, it is little resorted to after the termination of that process. It is to this *post-partem* use of the remedy we desire to call attention in the few thoughts that may be here hurriedly presented.

While the peculiar tendency in the action of ergot would seem to be to produce contraction of the muscular fibres of the gravid uterus, we think that we may venture the proposition that this *peculiar tendency is enhanced in the ratio of the increase of that tissue above the normal non-gravid uterus; and that the physiological action of ergot is to produce tonicity or contraction of the non-striated muscles wherever found.* Brown-Sequard has clearly shown that the action of ergot on the unstriped muscular coats of the arteries is to diminish their calibre and reduce the afflux of blood to the parts supplied by such vessels.

The action of this remedy, then, in arresting uterine hemorrhage would seem to be two-fold: first, by producing rigid contraction of the uterine walls by which the uterine vessels were compressed; and secondly, by diminishing the calibre of vessels conveying blood to that organ, by contracting their muscular coat. It is very evident that this two-fold action of ergot has much to do with the danger to the *fœtus in utero*, when administered for the purpose of promoting the process of parturition. The normal flow of blood through the uterine vessels is diminished, the maternal blood is cut off from the placental circulation, and the child becomes asphyxiated from failure to de-carbonize and properly oxygenize the fœtal blood in the placenta. The result is *fœtal suffocation.*

Another ground for venturing the opinion that the action of ergot is increased in the ratio of increase of development of the non-striated muscular tissue, is found in the susceptibility to its action in intra-mural myoid, myo-fibroid, and other uterine tumors, in which the muscular tissue is increased above that existing in the non-gravid uterus; and it is in just this class of cases we find most benefit from the administration of the remedy—the benefits resulting from its two-fold action alluded to above.

Some other histological or physiological facts, bearing upon uterine pathology in this connection, may not be out of place presented here. One of these is that the uterine veins present some peculiarities not found in any other portion of the venous system. In the construction of their walls the fusiform fibres of the non-striated muscles enter very largely; and another is, that the ganglionic nerves which supply the uterus divide themselves between the non-striated muscular tissue and the uterine arterial vessels, thus supplying all the fusiform fibres of the muscular walls of the uterus, the muscular coats of the arteries, and the muscular element of the venous vessels, with the same excito-motor or vaso-motor force, under the governing or modifyiug powers of the ganglionic nerve system, through which sympathetic relation is maintained with every portion of the maternal organism; relations more or less direct or intimate, but still essential to preserve a normal condition.

While we advance in civilization, in refinement, in mental culture, in the development of the brain and nerve system, at the expense—so to speak—of our more physical being, we find these relations between different portions of our organism the more easily disturbed—in the ratio of that advance—and must seek the means to preserve an equilibrium. These means will be found in those that best enable us to imitate nature; that will supply her deficiencies and restrain her excesses; that will strengthen her in her weak points, and control her action within normal bounds.

The great tendency to uterine disease, following parturition, particularly in the higher walks of life, where the brain and nerve system have been more highly developed at the expense of the physical organism (and especially among those who live a more sedentary life, and more especially with such as may fall under *malarial* impress, and suffer from portal engorgement), should awaken the profession to the necessity of resorting to any and every means at their command for warding off these diseases and the train of evils that follow in their wake.

While it will not be claimed that any one remedy at our command will accomplish this much desired end, it is claimed that much may be accomplished by closely watching and gently aiding the efforts of nature in gradually restoring the uterine organs to their primative state after parturition; and in this way many of these evils may be averted, and uterine disease prevented.

One of the great troubles, lying at the very foundation, in the long train of diseases that follow parturition, is *sub-involution*. What is it? A failure on the part of the uterus to resume its *non-gravid* dimensions; a failure, as Retzius would express it, of fatty degeneration; and this must carry us back to consider the healthful normal changes, and how they are produced; to see wherein the deficiency lies, and the indications to be met in aiding nature in carrying on the work.

In *primipara*, as a general rule, there is sufficient tone in the muscular tissue of the uterus to keep up a normal degree of post-partem contraction; and from that tonic contraction, as a like general rule, we have *no after-pains*. In subsequent labors, after the expulsion of the contents of the uterus, a rythmic contraction is set up, which continues with greater or less force—generally in the inverse ratio to the severity and duration of the labor—until the *tonic contraction* is well established, as in the primipara, when the rythmic action ceases, and with it the accompanying after-pains. During this rythmical contraction the venous sinuses are disgorged, emptying themselves into the cavity of the uterus, and ultimately, under a more powerful contraction, out of the cavity into the vaginal outlet. At the same time the contraction in the uterine walls compresses the uterine vessels, arteries and veins, forcing onward their contents, when fresh blood takes its place, and the uterine functional action is promoted, where, by reason of the stasis of the blood, it had been retarded.

In this rythmical action, the period of rest, *of relaxation*, is but the evidence of an atonic condition for the time being; the period of action, of contraction, is but an equal evidence of *reaction—tonicity* restored for the time being. Between these two conditions the organ vibrates, and the evidence is the after-pains, which attract most, the attention of the patient. We are aware of the fact that it is orthodox practice to arrest these after-pains by the administration of anodynes, per os or per anum. But what is the result? The "irritability" is allayed, the pain is relieved, the contractions are arrested, the patient is made more comfortable. But have we not thwarted nature in her efforts to carry on her work, instead of giving her aid in its accomplishment? Do we by this course establish the *tonic contraction of the uterus?* (It is admitted that opium is a nerve stimulant!) Several conditions may give rise to these rythmical contractions (after-pains), but the object of nature in each one is the establishment of a *tonic* contraction of the organ. They may arise from the retention of foreign substances (blood clots, membrane, placental fragments, exudations) in the cavity of the uterus; from coagula in the uterine sinuses; from extravasations or infractions in the uterine tissue; from a stasis of blood in the uterine vessels themselves, vitiated by reason of its condition of *stat*, or they may arise from a condition of *nerve paresis*, general or local (uterine). In the latter case the opium treatment would be eminently proper, but in the other cases presented it would seem that the indications of nature would point us to such remedies as would remove the cause, contract the uterine walls, and expel the interstitial exudations, the retained coagula of the sinuses and the contents of the cavity of what ever kind; contract and compress the vessels—both arterial and venous—and re-establish a normal flow of blood through the organ, by which its nutrition is kept up; its secretion and excretion secured; its absorbents and lymphatics relieved of over-work, and its *tonic contraction firmly established.*

To meet these indications, the physiological action of ergot would seem to point it out as a proper therapeutic agent; and the experience of many years in its use, in private practice, confirms this opinion. Opium (as above indicated), quinine, nux vomica, iron, and other adjuncts, may be called to its aid, as occasion may require, but for the pathological tendencies, in a large number of cases, the use of ergot, properly supported, will prevent many of the diseases that without its use invariably and inevitably follow the process of parturition.

It is not claimed as *new*, the use of ergot for the relief of after-pains. For this purpose it has been highly extolled by Crozat, Velpeau, Cazeaux, and others; but the leading idea with them was the expulsion of uterine contents, and the relief of the pain by the removal of the cause—that is, foreign substances in the cavity of the uterus. What is claimed here is, that by the use of ergot, *after delivery*, many of the uterine diseases that follow labor may be prevented by its administration in such doses and at such intervals as will secure the *tonic contraction of the uterine walls and the uterine vessels.* This condition of tonicity varies in different cases from the flaccidity or atony in which rapidly fatal hemorrhage follows, to the condition of tonic contraction in the primipara, where neither after-pains nor unpleasant sequelæ follow. Between these two extremes we find various grades of that condition, as that to which Leroux applied the term *humoral engorgement*, or Simpson the term *sub-involution*, and to which varied degrees, various other terms have been applied by different writers; a condition in which the parietes and vessels of the womb are over-distended, and from which they cannot relieve themselves, by reason of a want of sufficient activity or contractility in the muscular fibres entering into the composition of the vascular or uterine walls, or both.

Passing the first result of this antony—hemorrhage; and the second—after-pains (an effort at reaction—rhthmical), we find the uterine parietes engorged; their thickness increased; the volume magnified; the cavity effaced; its weight augmented by a superabundance of blood retained within the weakened vascular walls, in which the damages are increased by the hypostatic hyperæmia. What is the result of this condition of the uterus when allowed to continue? Apace with the disturbance in the circulation, must of necessity come disturbance in nutrition; modified, of course, by the condition of the blood. Through the thin walls of the blood-vessels exude first the more liquid parts of the blood, and we have infiltration of tissue; that infiltration reaching the uterine cavity, constitutes an increased lochia, or a leucorrhœal flow. A still greater degree of pressure or distension of the vascular walls, and *by diapedesis* the white and then the red blood-corpuscles leap through, modifying the exudations, or establishing other pathological conditions of the uterus. If the blood tissue is depraved, the flow increases, becoming both cause and effect. Should the blood be rich in fibrin-generating elements, that unite in the production of fibrin in the extra-vascular tissue, it may lead to a condition of more solid and permanent hypertrophy of the organ; to the production of fibroid deposits, or to other conditions not consistent with healthy action in the organ. These exudations or depositions may be variously modified by the physiological or pathological conditions of the system, resulting in the production of various forms of disease of which it is not our purpose to speak particularly at this time.

The object of this hurriedly written paper is to point to the fact that a very large number of the uterine diseases following labor may be traced back to the condition of *atony, want of contraction in the uterine, muscular, and vascular walls,* subsequent to parturition, and to assert the belief that in a very large per cent. of cases these sequelæ may be prevented and the diseases averted, by giving due attention to this *atony* of the uterine organ.

We are no strenuous advocate for the use of ergot during labor, for the purpose of hurrying up that labor, believing it to be, often times, dangerous, to both mother and child; but when, at the termination of that labor, we find the uterus exhausted, by reason of a pre-existing want of tone, by reason of exhaustion in the efforts of parturition, or from what ever cause, there is want of steady, firm, unyielding contraction of its *muscular* walls—upon pure physiological, as well as pathological, reasoning we may conclude that there is a like want of tone in its *vascular* walls; for its musculo-motor and vaso-motor nerves are the same. Such being the case, ergot becomes *par excellence the remedy.* We have no disposition to quarrel with our old friends, opium, camphor, hyoscyamus, and a host of like remedies, nor to discard them; they may be valuable adjuncts, as is also quinine, nux vomica, etc., alluded to above; but the one leading idea it is desired to promulgate here is, that *post-partem, uterine atony—muscular and vascular*—exists in most multiparous cases, in many primipara; that to the extent and duration of that *atony* there is a tendency to subsequent uterine disease of one form or another; that in the ratio that we overcome, timely, that atony, we will prevent the supervention of such disease; and that we have at our command to overcome that condition no more reliable remedy than ERGOT, and for the reasons assigned above—its well established physiological effects upon the non-striated muscular fibres of the uterus, and of its vascular walls, both arterial and venous.

One word with regard to the preparation. There is no one more useless drug than an unreliable preparation of ergot; there is no one in which its failure to act is so annoying to the practitioner; and there is no one from which we may expect a more prompt response than a reliable, genuine preparation. Hence it is of the utmost importance that he know absolutely that he is not relying upon a worthless article; and while it is not desired to make invidious distinctions, it may not be out of place to state that from *Squibbs' Fluid Extract* we have met the least disappointment.

